# Author Correction: A dynamic code for economic object valuation in prefrontal cortex neurons

**DOI:** 10.1038/ncomms16175

**Published:** 2017-11-23

**Authors:** Ken-Ichiro Tsutsui, Fabian Grabenhorst, Shunsuke Kobayashi, Wolfram Schultz

Nature Communications 7: Article number: 12554; DOI: 10.1038/ncomms12554 (2016); Published: 09 13 2016; Updated: 11 23 2017.

The previously published version of this Article contained an error in Figure 1. In panel b the *x* and *y* axis labels of the scatter graph were inadvertently inverted. This error has been corrected in both the PDF and HTML versions of the Article.

