# Supplementary material for: A dynamic code for economic object valuation in prefrontal cortex neurons
Source: Nat Commun. 2016 Sep 13;7:12554. doi: 10.1038/ncomms12554 (PMC5027248; doi:10.1038/ncomms12554)
Supplement: Supplementary Information — Supplementary Figures 1 - 8, Supplementary Tables 1 - 5 and Supplementary References [file ncomms12554-s1.pdf]

Supplementary Information

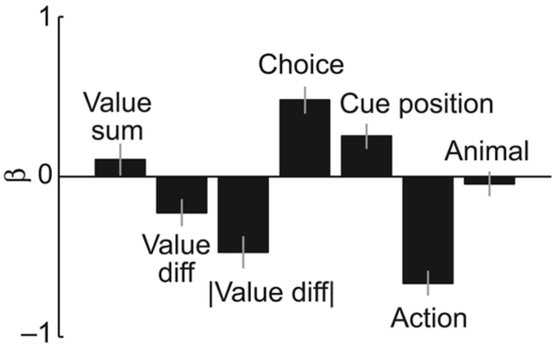

Supplementary Fig. 1. Influences on saccadic reaction time in choice period. Multiple regression of z-normalized saccadic reaction time on value sum (P = 0.286, t-test), signed value difference (P = 0.0077), unsigned value difference ($P = 1.8 \times 10^{-6}$), choice ($P = 1.6 \times 10^{-8}$), cue position (P = 0.001), action ($P < 1 \times 10^{-16}$), and animal identity (P = 0.571; 16,440 trials, *d.f.* = 16,432).

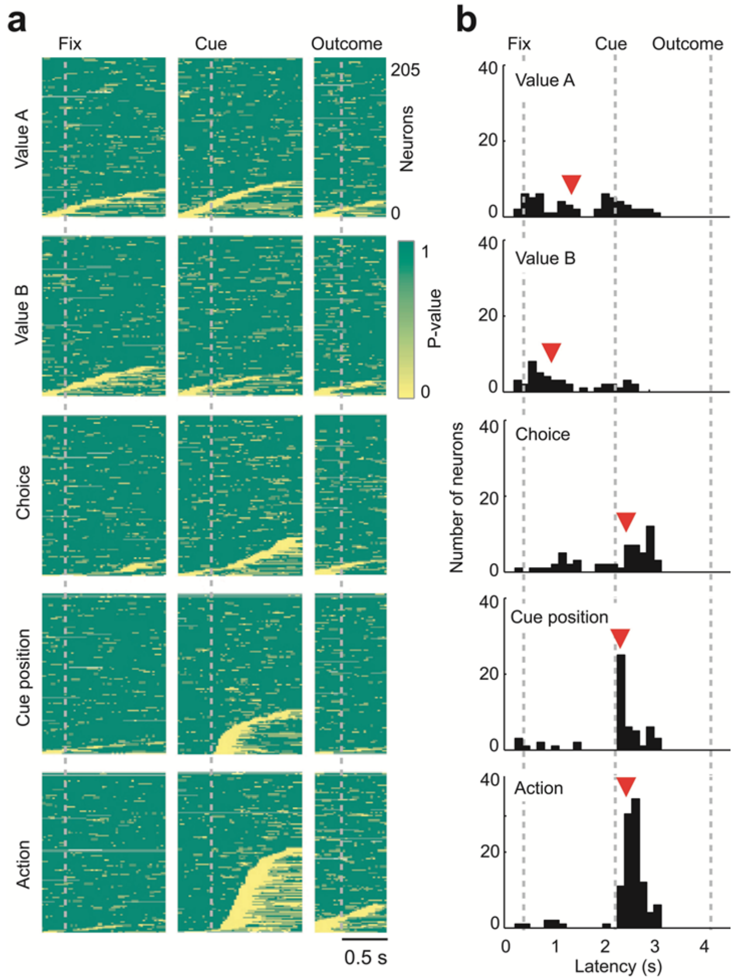

Supplementary Fig. 2. Temporal dynamics of value encoding and other task variables. a. Statistical *P*-values for value A, value B, choice, cue position and action in all neurons, obtained from sliding window multiple regressions aligned to fixation, cue and outcome events. Data in each row are from a single neuron. Color code indicates *P*-value. Neurons are sorted from bottom to top within each panel according to coding latency. For clarity, *P*-values > 0.05 were set to 1. Isolated white areas indicate periods in which a neuron was silent. **b**. Latency distributions based on analysis in **a**. Compared to value encoding, a lower but significant number of neurons encoded the animal's upcoming choices before cue appearance, as early as 1 s before cues (**a**), although mean onset latency for choice coding followed cue appearance (**b**). The number of choice-predictive neurons in the pre-cue phases was significantly higher than expected by chance ($P < 10^{-7}$, binomial test) and significantly higher ($P < 0.05$, z-test for dependent samples) than any neurons showing false positive pre-cue effects for actions or cue position (both < 5%). Thus, the presence of choice-predictive responses at early trial stages provided evidence for an object-based decision process that took place before the animal knew where to direct its gaze to express the object choice. Consistent with previous studies[1,2], a large number of DLPFC neurons encoded spatial cue position and the animal's action in task periods following cue appearance (**a,b**).

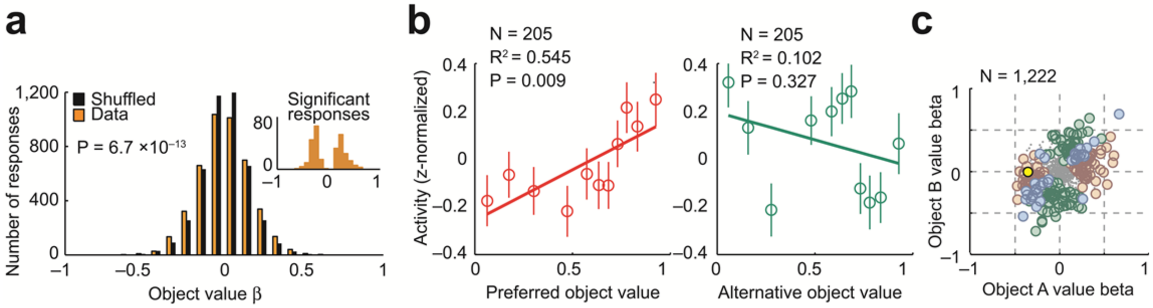

Supplementary Fig. 3. Population characteristics of value coding. a. Distribution of regression coefficients for object value based on actual data (orange) and trial-shuffled data (black) of fixed window analysis. The distribution based on actual data was significantly different from shuffled data (P = 6.7 × 10^{-13}, Kolmogorov-Smirnov test) and shifted towards larger positive and negative values. Inset shows distribution of significant responses. **b**. Object-specificity of value coding. Linear regression of population activity (205 neurons) on object value for preferred and alternative object. For each neuron with a significant value effect (of either object A or B), we selected the task periods with the highest value correlation for each object and then plotted the activity from each period against object value. The preferred object value in the plot corresponds to the object with the highest value correlation; the alternative object value corresponds to the other object value for which also plotted data from the period with the highest value correlation for that object. Thus, the choice of best interval for object value coding was done independently for the two objects so that the plot is not biased in favor of either object. Data points indicate means of 11 equally populated value bins ± s.e.m. **c.** Standardized regression coefficients for all task-related responses (red, significant object A value; green, significant object B value; blue: both value coefficients significant; grey: neither value coefficient significant). Yellow data point: example neuron from Fig. 2. Data in all plots are taken across all task periods.

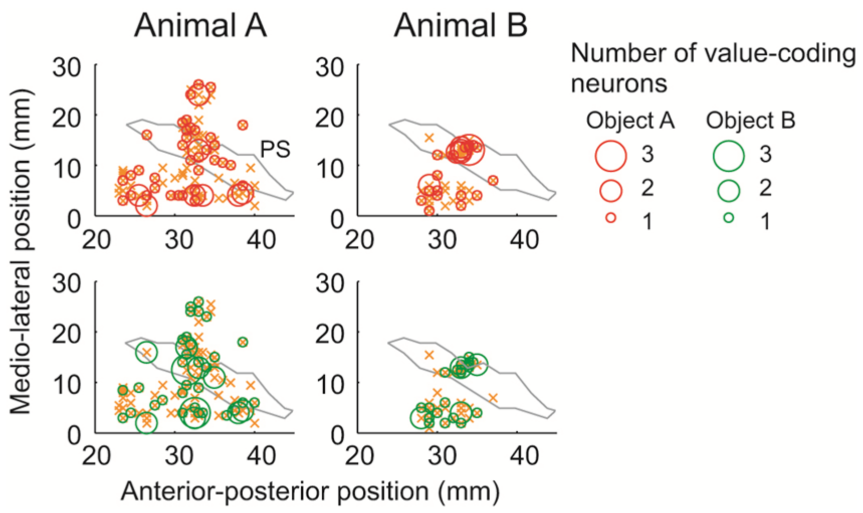

Supplementary Fig. 4. Anatomical location of recording sites. Anterior-posterior position was defined with respect to inter-aural line. Orange crosses indicate locations for all recorded neurons. PS, approximate position of principal sulcus.

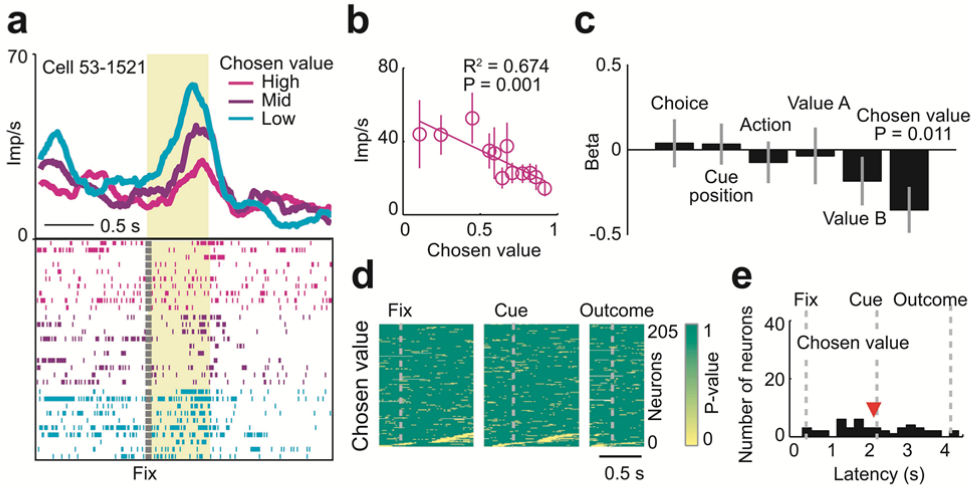

Supplementary Fig. 5. Neuronal coding of chosen value. Rather than coding object value, a neuronal response might reflect 'chosen value', that is, the value of the chosen object irrespective of its identity. Chosen value coding in different brain structures has been observed for objects[3,4] and actions[5] and is usually interpreted as a post-decision signal suitable for reward evaluation and learning but not as input for choice. Although most value responses in the present experiment did not reflect choice, we tested specifically for chosen value using additional regressions with chosen value as covariate alongside object value. **a**. A single DLPFC neuron encoding chosen value. Peri-event time histogram of impulse rates, aligned to cue onset, sorted into terciles of chosen value. Raster display: ticks indicate impulses, rows indicate trials; grey dots indicate event markers for fixation spot onset. Fixation activity reflected the value of the chosen object. Yellow shaded period was used for analysis. **b**. Linear regression of fixation-period impulse rate on chosen value (means of 13 equally populated value bins ± s.e.m, *d.f.* = 11). **c**. Coefficients obtained from fitting a multiple linear regression model to fixation-period impulse rate. Only chosen value explained a significant proportion of variance in impulse rate (P = 0.011, t-test, N = 68 trials, *d.f.* = 60). **d**. Statistical P-values for chosen value across all neurons, obtained from sliding window multiple regression. **e**. Latency distribution based on data in **d**. Red arrowhead indicates median. A moderate number of task-related responses were directly related to chosen value (91/1222, 7.4%). Their temporal dynamics resembled those of choice-coding responses: they occurred in some cases before cue appearance but typically not as early as object value signals (**d,e**). Critically, including the chosen value covariate in the regression model had little effect on the number of object value responses (265 responses, compared to 273 in our main model). Thus, object value and chosen value explained distinct portions of variance in DLPFC neuronal responses.

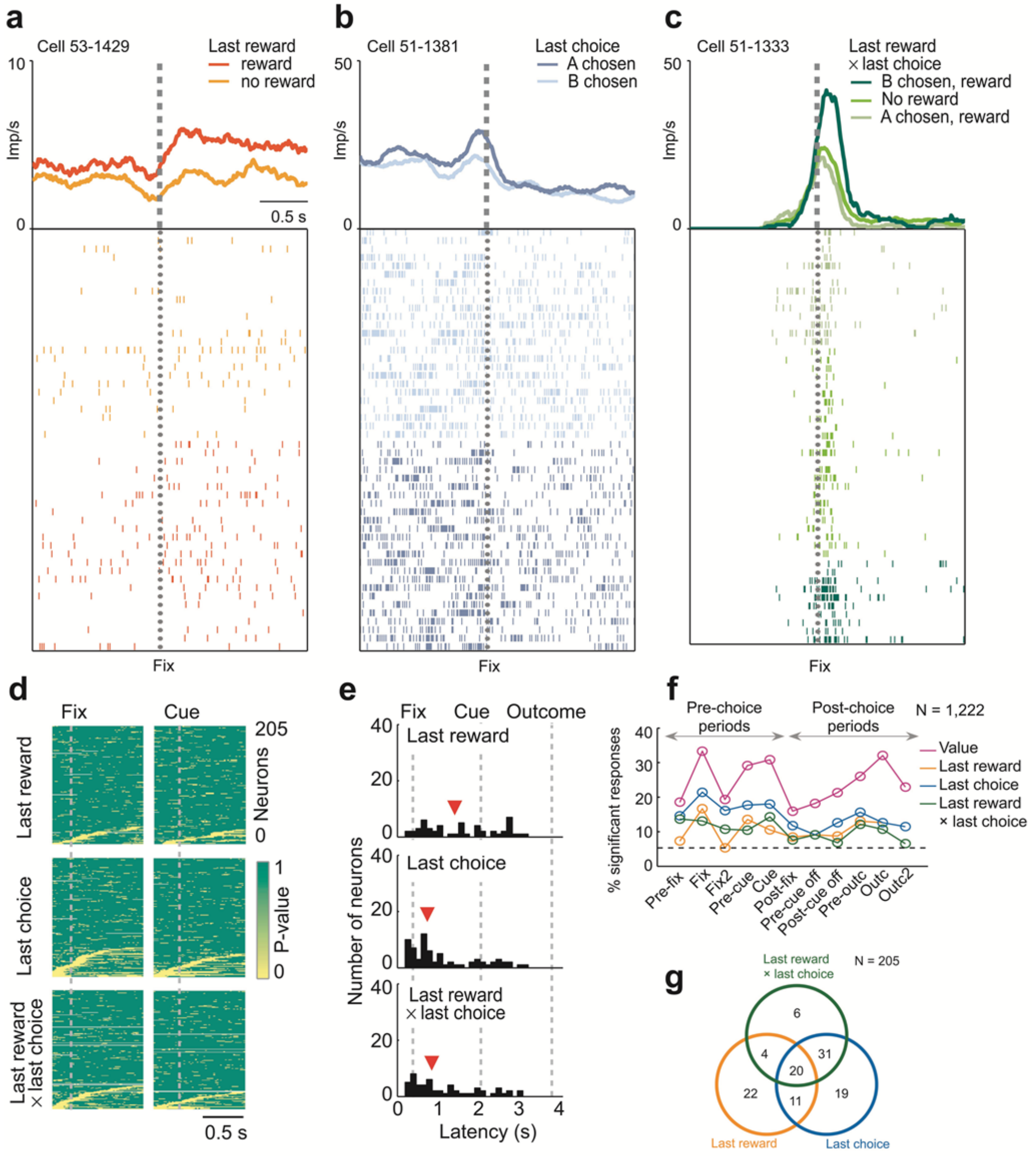

Supplementary Fig. 6. Coding of reward and choice history in DLPFC neurons. **a.** A single DLPFC neuron whose fixation activity reflected last-trial reward history. This effect was not explained by current-trial variables which were included as covariates in the multiple regression. **b.** A single DLPFC neuron whose pre-fixation activity reflected last trial choice history. **c.** A single DLPFC neuron whose fixation activity reflected last trial reward × choice interaction; activity was strongest if object B was chosen and rewarded on the last trial. **d.** Statistical *P*-values for last-trial parameters across all neurons obtained from sliding multiple regression. **e**. Latency distributions based on analysis in **d**. **f.** Percentages of task-related responses with significant value and history coefficients in different trial periods. **g.** Summary of neurons with significant coding of reward history, choice history, reward × choice history and their conjunctions, obtained from sliding window regression.

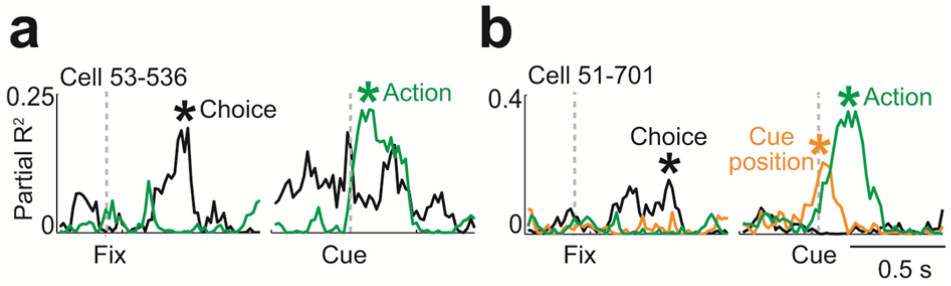

Supplementary Fig. 7. Choice-to-action coding transitions in two DLPFC neurons. a. A single DLPFC neurons showing choice-to-action transition, similar to DLPFC neurons recently reported in an economic choice task[4]. Coefficients of partial determination (partial R^2) from a sliding window multiple regression analysis. **b**. A single DLPFC neurons showing a transition from choice to cue position and action.

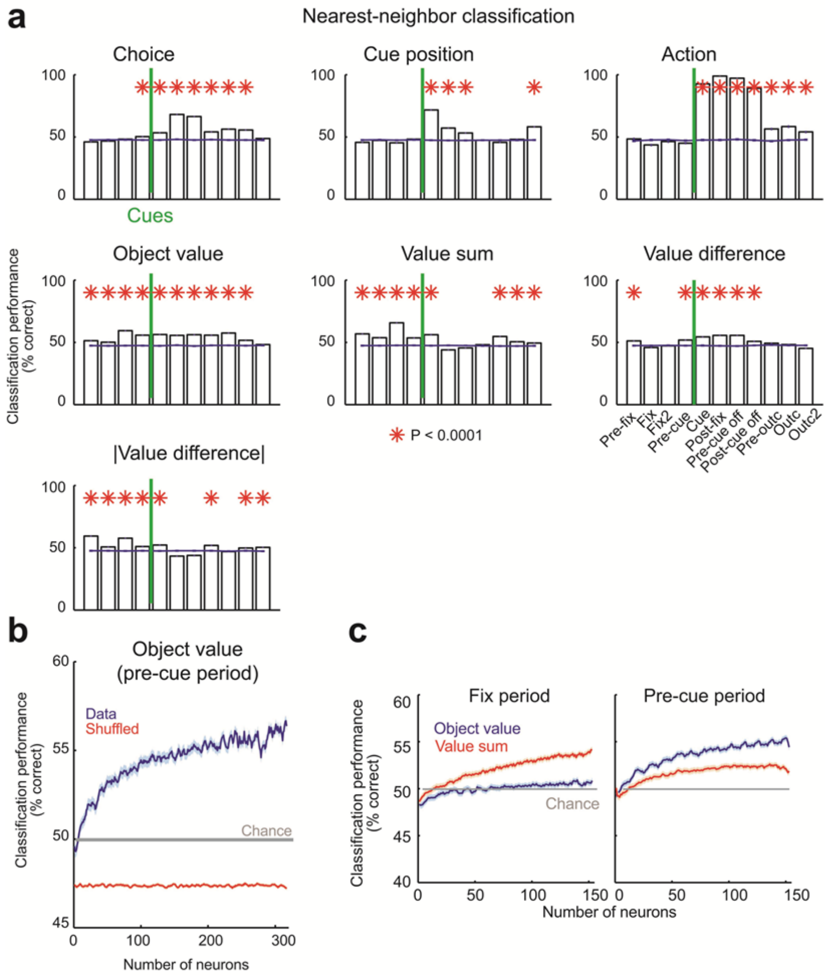

Supplementary Fig. 8. Population decoding with nearest-neighbor classifier. a. Performance of a nearest neighbor classifier in decoding task variables in different task periods. Performance was measured as cross-validated classification accuracy (% correct, mean ± s.e.m.) based on single-trial data from all DLPFC neurons that met inclusion criteria for decoding (*N* = 166). Normalized impulse rates from independently recorded neurons were aggregated into pseudo-populations. The grey line in each plot indicates mean (± s.e.m) decoding performance from trial-shuffled data. Red asterisks indicate that decoding accuracy significantly exceeded shuffled decoding (rank-sum test). **b.** Nearest-neighbor decoding performance as a function of neuron number for object value in pre-cue period. **c.** Nearest-neighbor decoding performance as a function of neuron number for object value and value sum in fixation period (left) and pre-cue period (right).

Supplementary Table 1. Numbers of neurons (and percentages) showing specific effects with fixed window analysis.

	**Animal A**	**Animal B**	**Both**
Total	140	65	205
Value[1]	76 (54%)	43 (66%)	119 (58%)
Value A[2]	51 (67%)	30 (70%)	81 (40%)
Value B[2]	53 (70%)	28 (65%)	81 (40%)
Achosen[1]	58 (41%)	25 (38%)	83 (41%)
ALeft[1]	52 (37%)	23 (35%)	75 (37%)
Left/right[1]	83 (59%)	35 (54%)	118 (58%)

[1] Percentages calculated with respect to neurons in row 'Total' of the same column
[2] Percentages calculated with respect to neurons in row 'Value' of the same column

Supplementary Table 2. Numbers of neurons (and percentages) showing specific effects with sliding window analysis.

	Animal A	Animal B	Both
Total	140	65	205
Value[1]	70 (50%)	41 (63%)	111 (54%)
Value A[2]	49 (70%)	30 (73%)	79 (39%)
Value B[2]	42 (60%)	22 (54%)	64 (31%)
Achosen[1]	47 (34%)	27 (42%)	74 (36%)
ALeft[1]	46 (33%)	25 (38%)	71 (35%)
Left/right[1]	84 (60%)	35 (54%)	119 (58%)

[1] Percentages calculated with respect to neurons in row ‘Total’ of the same column
[2] Percentages calculated with respect to neurons in row ‘Value’ of the same column

Supplementary Table 3. Numbers of neuronal responses (and percentages) showing specific effects with fixed window analysis.

Variable	Effect	Animal A	Animal B	Both
Total	Task-related	847	375	1222
Value A[1]		85 (10%)	51 (14%)	136 (11%)
	-other[2,3]	39 (46%)	24 (47%)	63 (46%)
	+other[3,4]	46 (54%)	27 (53%)	73 (54%)
	+Value B[4,5]	21 (46%)	13 (48%)	34 (47%)
	+Achosen	14 (30%)	10 (37%)	24 (33%)
	+Aleft	11 (23%)	4 (15%)	15 (21%)
	+Left/Right	16 (35%)	11 (41%)	27 (37%)
Value B[1]		93 (11%)	44 (12%)	137 (11%)[1]
	-other[2,3]	47 (51%)	20 (45%)	67 (49%)
	+other[3,4]	46 (49%)	24 (55%)	70 (51%)
	+Value A[5]	21 (46%)	13 (54%)	34 (49%)
	+Achosen	11 (24%)	4 (17%)	15 (21%)
	+Aleft	10 (22%)	4 (17%)	20 (29%)
	+Left/Right	19 (41%)	10 (42%)	32 (46%)
Value A & B[1]		21 (2%)	13 (4%)	34 (3%)[1]
	Value Sum[3]	11 52(%)	8 (62%)	19 (56%)
	Value Diff[3]	10 (48%)	5 (38%)	15 (44%)
Non-value[1]		690 (81%)	293 (78%)	983 (80%)[1]
	Achosen[3]	84 (12%)	35 (12%)	119 (12%)
	Aleft[3]	54 (8%)	27 (9%)	81 (8%)
	Left/Right[3]	138 (20%)	66 (23%)	204 (21%)

[1] Percentages calculated with respect to responses in row ‘Total’ of the same column

[2] Value coding without coding of additional variables

[3] Percentages calculated with respect to responses in row ‘Total’ row of the same column

[4] Value coding jointly with coding of additional variables

[5] Percentages calculated with respect to responses in row ‘-other’ of the same column

Supplementary Table 4. Numbers of neurons (and percentages) showing specific effects with stepwise regression.

	**Animal A**	**Animal B**	**Both**
Total	140	65	205
Value[1]	99 (71%)	49 (75%)	148 (73%)
Value A[2]	63 (45%)	27 (42%)	90 (44%)
Value B[2]	47 (34%)	26 (40%)	73 (36%)
Chosen	44 (31%)	25 (38%)	69 (34%)
Achosen[1]	63 (45%)	25 (38%)	88 (43%)
ALeft[1]	53 (38%)	21 (32%)	74 (36%)
Left/right[1]	87 (62%)	39 (60%)	126 (61%)

[1] Percentages calculated with respect to neurons in row 'Total' of the same column

[2] Percentages calculated with respect to neurons in row 'Value' of the same column

Supplementary Table 5. Numbers of neurons (and percentages) showing specific effects with sliding window analysis (extended model).

	**Animal A**	**Animal B**	**Both**
Total	140	65	205
Value[1]	80 (57%)	39 (60%)	119 (58%)
Value A[2]	54 (39%)	28 (43%)	82 (40%)
Value B[2]	55 (39%)	26 (40%)	81 (40%)
Last reward[1]	35 (25%)	22 (34%)	57 (28%)
Last choice[1]	49 (35%)	32 (49%)	81 (40%)
Last reward × last choice[1]	39 (28%)	22 (34%)	61 (30%)
Achosen[1]	49 (35%)	25 (39%)	74 (36%)
ALeft[1]	45 (32%)	22 (34%)	67 (33%)
Left/right[1]	82 (68%)	31 (48%)	113 (55%)

[1] Percentages calculated with respect to neurons in row ‘Total’ of the same column
[2] Percentages calculated with respect to neurons in row ‘Value’ of the same column

References

1. Funahashi, S. Space representation in the prefrontal cortex. *Progress in neurobiology* **103**, 131-155 (2013).
2. Hoshi, E. & Tanji, J. Area-selective neuronal activity in the dorsolateral prefrontal cortex for information retrieval and action planning. *Journal of neurophysiology* **91**, 2707-2722 (2004).
3. Padoa-Schioppa, C. & Assad, J.A. Neurons in the orbitofrontal cortex encode economic value. *Nature* **441**, 223-226 (2006).
4. Cai, X. & Padoa-Schioppa, C. Contributions of orbitofrontal and lateral prefrontal cortices to economic choice and the good-to-action transformation. *Neuron* **81**, 1140-1151 (2014).
5. Lau, B. & Glimcher, P.W. Value representations in the primate striatum during matching behavior. *Neuron* **58**, 451-463 (2008).
